# *Ophiorrhiza guizhouensis* (Rubiaceae), a new species from Guizhou Province, southwestern China

**DOI:** 10.3897/phytokeys.95.22506

**Published:** 2018-03-06

**Authors:** Chuan-Dong Yang, Xuan-Ze He, Guang-Qian Gou

**Affiliations:** 1 Tongren University, Tongren 554400, Guizhou, China; 2 Fanjingshan National Nature Reserve Administration of Guizhou, Tongren 554400, Guizhou, China; 3 College of Life Sciences, Guizhou University, Guiyang 550025, Guizhou, China

**Keywords:** *Ophiorrhiza*, Guizhou, China, new taxa, distyly

## Abstract

In this study, *Ophiorrhiza guizhouensis*, a new species of Rubiaceae from south-western China, is described and illustrated. The new species is morphologically similar to *O. japonica*, but differs from the latter by having terete stems which are densely hirtellous, usually persistent ciliate stipules with well-developed colleters inside the base of the stipule, shorter corolla tubes and shorter stamens and styles.

## Introduction

*Ophiorrhiza* L. is a member of tribe Ophiorrhizeae, subfamily Rubioideae, Rubiaceae (Bremer and Eriksson 2009), including more than 300 species worldwide (WCSPF 2017). The genus is mainly distributed in wet tropical forests of South-East Asia, extending to Australia, New Guinea and the Pacific Islands (Darwin 1976, Chen and Taylor 2011). *Ophiorrhiza* is a taxonomically difficult genus and is poorly known in South-East Asia (Chen and Taylor 2011). In China, 68 species, including 47 endemics, are recorded (Chen and Taylor 2011, Deng and Huang 2012, Wu et al. 2017a, b) and most of them are distributed in the region south of the Changjiang River, especially the provinces of Yunnan and Guangxi (Lo 1999).

During field work in north-eastern Guizhou, China, some specimens of *Ophiorrhiza* were collected. After carefully examining the specimens and living materials and reviewing the relevant literature (Lo 1990, 1999, Chen and Taylor 2011), it was concluded that the newly found plants represented an undescribed species. Here, the new species is described and illustrated.

## Materials and methods

Specimens were collected during February 2017. Additionally, some flowers were also collected and preserved in FAA for subsequent observations. The photographs were taken in the field. Morphological observations and measurements of the new species were carried out based on living plants, dry specimens and preserved materials.

## Taxonomy

### Ophiorrhiza guizhouensis

Taxon classificationPlantaeORDOFAMILIA

C.D.Yang & G.Q.Gou sp. nov.

urn:lsid:ipni.org:names:60476091-2

Figs 1 , 2

#### Diagnosis.

Similar to *O. japonica* Blume, but distinguished from the latter by the terete, densely hirtellous stems (vs. stems subterete to slightly compressed, glabrous or with 2 hirtellous or pilosulous lines), the usually persistent and ciliate stipules (vs. caducous glabrescent stipules), the well-developed colleters inside the base of stipule (vs. without colleters), the shorter corolla tubes (8–9 mm vs. 9–14 mm) and the shorter stamens and styles (longistylous flower: 2.5–2.7 mm and 7–8 mm vs. 4.5–5.5 mm and 9–11 mm; brevistylous flower: 3.6–4.2 mm and ca. 2.5 mm vs. 4.5–5 mm and ca. 3 mm).

#### Type.

CHINA. Guizhou: Tongren, Jiangkou County, Dewang Town, Miaowangpo, in broad-leaved forest, elevation 868 m, 27°46'31.87"N ,108°33'0.84"E, 19 Feb. 2017, *C.D. Yang 092* (Holotype: GACP!; Isotypes: GACP!, PE!, KUN!)

**Figure 1.**
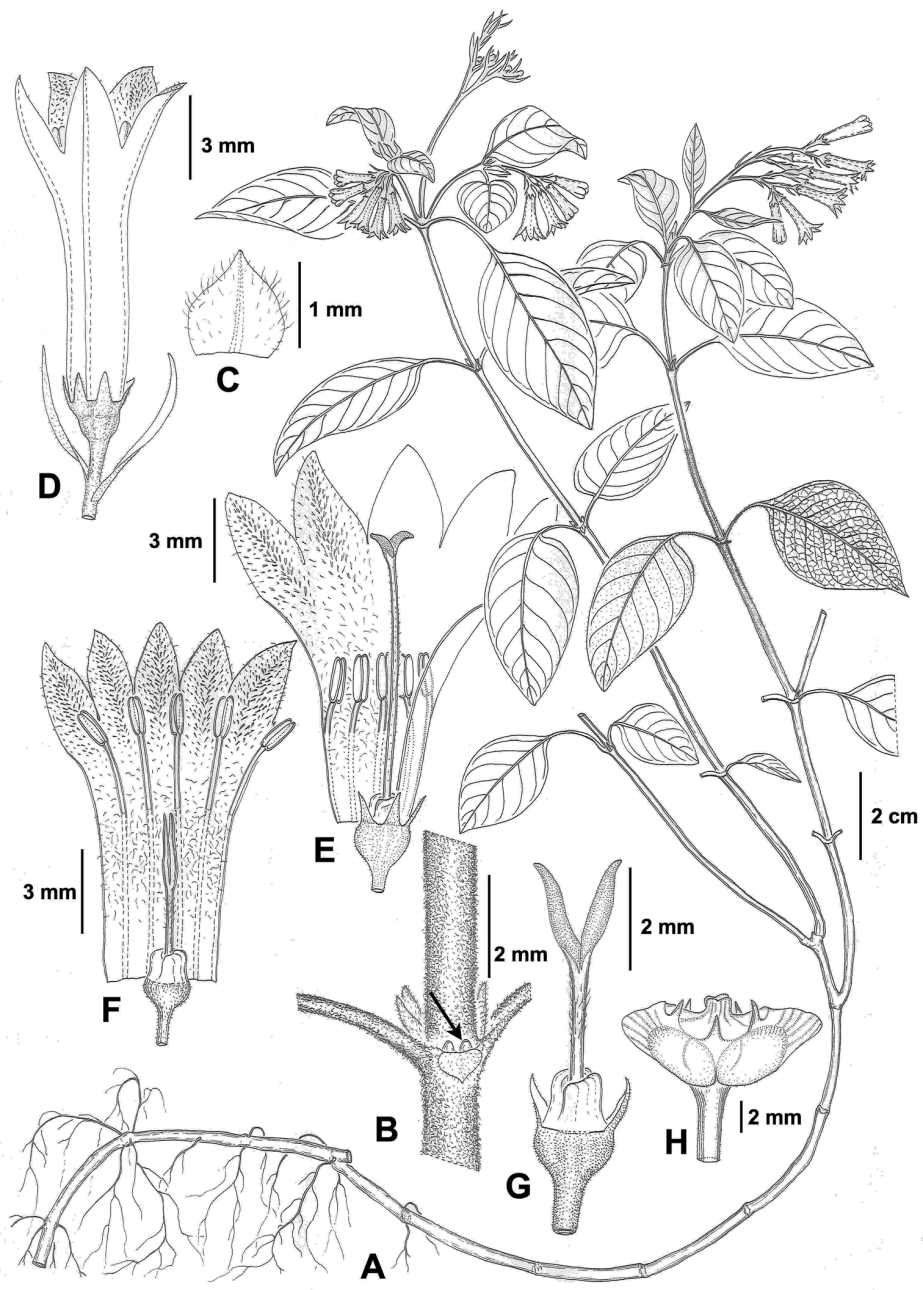
*Ophiorrhiza guizhouensis* **A** habit **B** stem, showing a stipule with two colleters (arrow) at the base inside **C** stipule, abaxial view **D** flower and bracts **E** dissected longystylous flower, showing stamen and style position **F** dissected brevistylous flower, showing stamen and style position **G** pistil of brevistylous flower **H** fruit. Drawn by Xiao-Yu Wang from the holotype.

#### Description.

Perennial herbs, ascending, 40–60 cm tall; stems branched, terete, the lower stems prostrate, rooting from the node, the upper stems erect, densely hirtellous. Leaves in subequal pairs; petiole 1.3–3 cm, hirtellous; leaf blade drying papery, ovate, broadly ovate or elliptic, 3–6 cm × 1.8–3 cm, drying brown adaxially, purple abaxially, sparsely hirtellous on both surfaces, densely hirtellous on the midrib abaxially; base cuneate to obtuse, apex acute to obtuse; margin flat to crisped; secondary veins 5–8 on each side of the midrib; stipules persistent, rarely caducous, ovate-triangular, 1.3–1.6 mm × 0.9–1.2 mm, glabrescent adaxially, puberulent abaxially, margin ciliate, with two well-developed colleters inside the base of stipules, apex acute to acuminate. Inflorescence terminal, congested-cymose to cymose, 5–30-flowered, hirtellous; peduncle 1–2 cm, densely hirtellous. Bracts linear, 4–5 mm × 0.8–1.1 mm, glabrescent adaxially, puberulent abaxially, persistent. Flowers distylous. Pedicel 1–3 mm long, pubescent. Calyx densely puberulent; hypanthium subturbinate, 0.8–1.1 mm long, 5-ribbed; lobes triangular, 1–1.5 mm long. Corolla purple in bud, white at anthesis, drying pink, funnelform to tubular-funnelform, longitudinally winged, glabrous outside; tube 8–9 mm long, villous inside; corolla lobes triangular to ovate, ca. 4 × 2 mm, villous inside, dorsally ridged. Longistylous flower: stamens included, inserted in middle lower part of the corolla tube; filaments ca. 1 mm long; anthers linear, 1.4–1.7 mm long; style filiform, 7–8 mm long, sparsely puberulent; stigmas 2-lobed, lobes ovate, ca. 1.2 mm long, glabrous. Brevistylous flower: stamens exerted, inserted at the middle of the corolla tube; filaments 1.8–2.1 mm long; anthers oblong linear, subequal to filament; style filiform, ca. 2.5 mm long, sparsely puberulent; stigmas deeply 2-lobed, linear-lanceolate, ca. 2 mm long, glabrous. Capsules obcordate in outline, 3.5–4.5 × 6.5–8 mm, subglabrous. Seeds small, angular, numerous, pale yellow to brown.

#### Phenology.

Plants were observed in full bloom on 19 February 2017. It can be expected that flowering time of the new species is from January to March; fruiting time needs further observations.

#### Distribution and habitat.

*Ophiorrhiza guizhouensis* is currently only known from Jiangkou County, Guizhou, south-western China, where at least 300 individuals are found. The species grows in evergreen broad-leaved forest or bamboo forest at elevations between 850–1,000 m, along with *Chimonobambusa angustifolia* C.D. Chu & C.S. Chao, *Lindera communis* Hemsl., L. pulcherrima var. hemsleyana (Diels) H.B. Cui, *Clematis henryi* Oliv. and *Ophiopogon bodinieri* H. Lév.

#### Etymology.

The specific epithet refers to Guizhou, a province of south-western China in which the new species was collected.

#### Preliminary conservation status.

The new species is currently only known from the type locality. More explorations are needed to fully understand its distribution and to assess its conservation status. Based on the available data, the new species is treated as “Data Deficient (DD)” according to the IUCN Red List Categories and Criteria guidelines (IUCN 2012).

**Figure 2.**
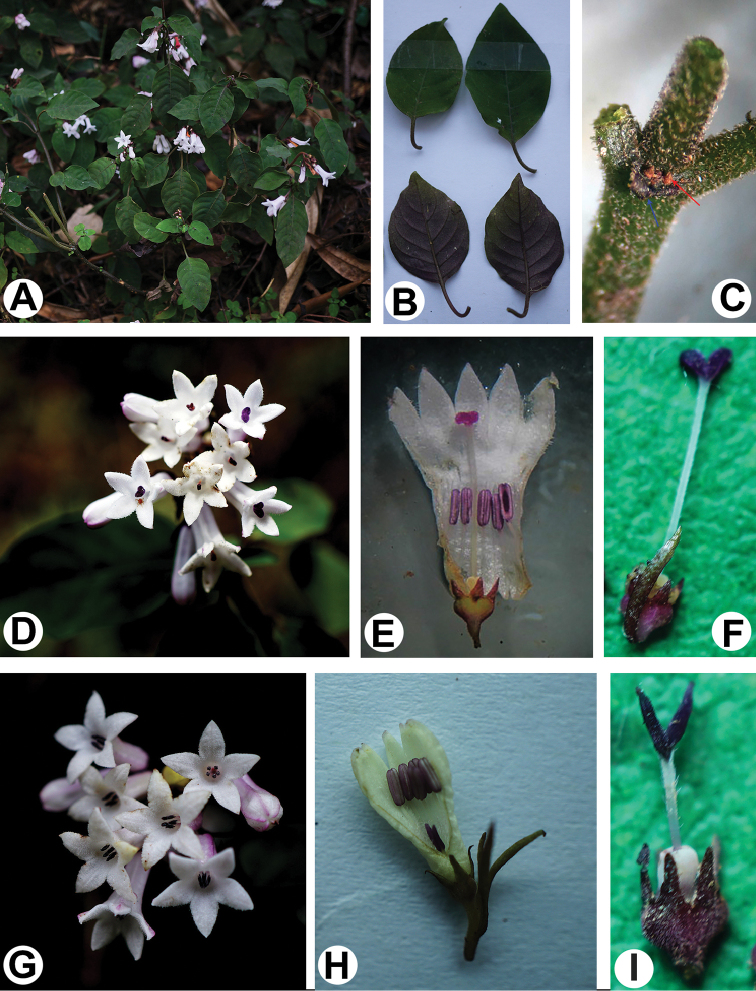
*Ophiorrhiza guizhouensis* **A** habit **B** leaves **C** node, showing persistent stipule (blue arrow) and colleters (red arrow), also showing stem pubescence **D** inflorescence, showing longistylous flowers **E** dissected longistylous flower **F** pistil of longistylous flower **G** inflorescence, showing brevistylous flowers **H** dissected brevistylous flower **I** pistil of brevistylous flower.

## Supplementary Material

XML Treatment for Ophiorrhiza guizhouensis
